# Ultrasound-guided erector spinae plane block for postoperative analgesia: a meta-analysis of randomized controlled trials

**DOI:** 10.1186/s12871-020-00999-8

**Published:** 2020-04-14

**Authors:** Jiao Huang, Jing-Chen Liu

**Affiliations:** Department of Anesthesiology, First Affiliated Hospital of Guangxi Medical University, 6 Shuangyong Road, Nanning, 530021 Guangxi Zhuang Autonomous Region People’s Republic of China

**Keywords:** Erector Spinae plane block (ESPB), Postoperative analgesia, Regional blockade, Opioid, Pain score

## Abstract

**Background:**

Ultrasound-guided Erector Spinae Plane Block (ESPB) has been increasingly applied in patients for postoperative analgesia. Its effectiveness remain uncertain. This meta-analysis aimed to determine the clinical efficacy of ultrasound-guided ESPB in adults undergoing general anesthesia (GA) surgeries.

**Methods:**

A systematic databases search was conducted in PubMed, Embase, and the Cochrane Library for randomized controlled trials (RCTs) comparing ESPB with control or placebo. Primary outcome was iv. opioid consumption 24 h after surgery. Standardized mean differences (SMDs) and risk ratios (RRs) with 95% confidence intervals (CIs) were calculated with a random-effects model.

**Results:**

A total of 12 RCTs consisting of 590 patients were included. Ultrasound-guided ESPB showed a reduction of intravenous opioid consumption 24 h after surgery (SMD = − 2.18; 95% confidence interval (CI) -2.76 to − 1.61,*p* < 0.00001). Considerable heterogeneity was observed (87%). It further reduced the number of patients who required postoperative analgesia (RR = 0.41,95% CI 0.25 to 0.66,*p* = 0,0002) and prolonged time to first rescue analgesia (SMD = 4.56,95% CI 1.89 to 7.22, *p* = 0.0008).

**Conclusions:**

Ultrasound-guided ESPB provides effective postoperative analgesic in adults undergoing GA surgeries.

## Background

Ultrasound-guided Erector Spinae Plane Block (ESPB) is a novel regional anesthesia technique that local anesthetic (LA) injection is performed into the fascial plane situated between the transverse process of the vertebra and the erector spinae muscles it is considered a relatively safe simple technique to perform [1, 2]. Followed by first description by Forero et al. [1] in 2016, it has been demonstrated successfully to provide analgesia in thoracic and thoracoabdominal surgeries [3, 4] However, the use of ultrasound-guided ESPB remained controversial. Recently, several randomized controlled trials (RCTs) [5–7] on this topic have been published, but the determine conclusions cannot be established owing to the modest sample size of these RCTs. We therefore conducted a meta-analysis to examine the efficacy of ultrasound-guided ESPB among adults undergoing general anesthesia (GA) surgery. Our primary outcome was intravenous opioid consumption 24 h after surgery. Secondly outcomes included pain scores, number of patients who need rescue analgesia, time to first rescue analgesic and postoperative nausea or vomiting (PONV).

## Methods

### Literature search and selection criteria

This systematic review and meta-analysis of RCTs was reported abiding by the Preferred Reporting Items for Systematic Reviews and Meta Analyses (PRISMA) statement [8] and it was conducted base on the statement of the Cochrane Handbook for Systematic Reviews of Interventi*ons* [9]*.* No formal protocol was registered for this meta-analysis.

PubMed, EMBASE, and the Cochrane Library were searched from inception to August 2019 with no language restriction. The search terms used were:(‘erector spinae plan block’ OR ‘erector spinae block’ OR ‘erector spinae plan blocks’ OR ‘erector spinae blocks’). The bibliographies of included trials were also manually searched for any eligible trials missed by the electronic search. This process was conducted iteratively until no extra reference could be verified.

Two of us independently performed the preliminary data search, after removing duplicate references, the titles and abstracts were screening for the eligible trials. We included all RCTs in adults who were undergoing GA surgery with the intervention of ultrasound-guided ESPB Trials were excluded for the following criteria: animal or cadaveric studies; reviews; did not report opioid consumption or pain scores as an outcome; Any discrepancies were resolved by discussion with coauthors.

### Data extraction and quality assessment

Data collection was performed by two authors (JH and JCL). The following information was collected from each eligible trial: first author, publication year, patient number, patient characteristics, American Society of Anesthesiologists (ASA) physical status, surgical procedure, ESPB group (position, dosage and concentration), control group (placebo or no invention). Extracted data were entered into a predefined standardized Excel (Microsoft 6 Corporation, USA) file.. For continuous data, we calculated mean and SD, if not provided, median and interquartile range were seen as means and standard deviation (SD) approximately as follows: the median was considered equal to the mean, and the SD was calculated as the interquartile range divided by 1.35 [10]. Any uncertainty arose were figured out though a consensus achieved.

Two authors (JH and JCL) evaluated the methodological quality of the trials according to the Cochrane risk-of-bias tool [11]. Each item was categorized as having a ‘low’, ‘unclear’, or ‘high’ risk of bias. Any uncertainty arose were resolve by discussion between two researches until a consensus was achieved.

### Statistical analysis

The relative risks (RRs) and standardized mean differences (SMDs) with 95% confidence intervals (CIs) were calculated. A random effects model was selected to acquire the most conservative effects estimate. An I^2^ statistic of 25–50% were defined as low heterogeneity, an I^2^ statistic of 50–75% were described as moderate heterogeneity, and those with an I^2^ statistic of > 75% were considered as high heterogeneity [12], The heterogeneity was substantial when an I^2^ value was over 50%. Subgroup analysis was conducted based on additional analgesia (patient-controlled analgesia device (PCA) versus not PCA). Publication bias was evaluated using funnel plots. Statistical analyses were calculated using the Review Manager Version 5.3 (Nordic Cochrane Centre, Cochrane Collaboration).

## Results

### Study identification and characteristics

**Fig. 1:**
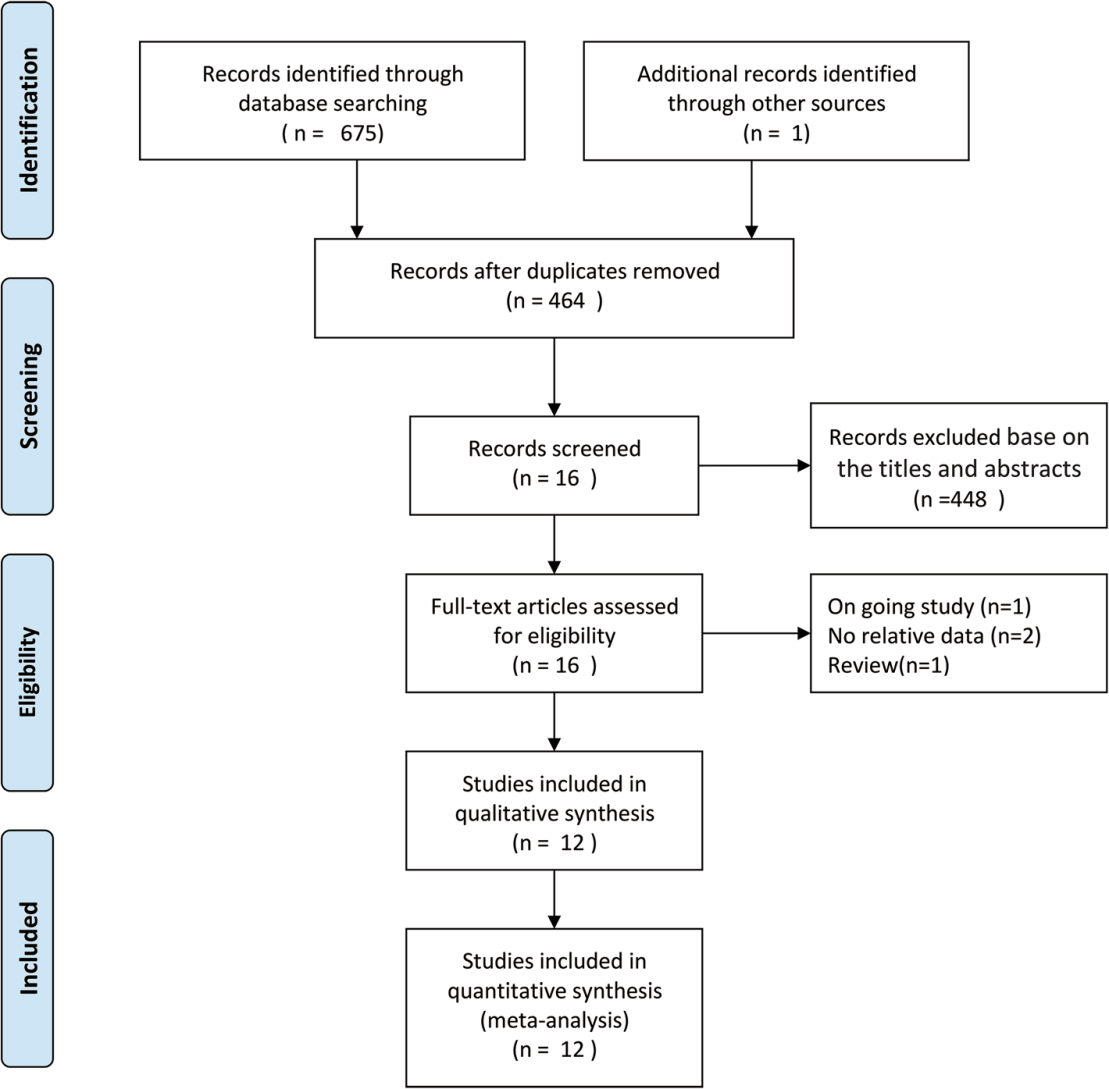
PRISMA flow diagram showing literature search results

A total of 675 studies were obtained by the literature search. One further citations were found by hand searching. 212 records were excluded for duplicate studies and a further 448 records removed by screening titles and abstracts. 16 full text publications remained were scrutinized for conclusive identified. 4 of them were excluded because 2 did not report data of interest [13, 14], one was currently ongoing study [15],one was review article [16].Finally,12 RCT [5–7, 17–25] satisfied our inclusion criteria. A flowchart of the literature search is shown in (Fig. 1).

All RCTs included in this meta-analysis were published between 2018 and 2019, with a total of 490. The main characteristics of the 12 RCTs included are presented in Table 1.

**Table 1:** Characteristics of included studies

	No. Of patients	Surgical procedure	ASA	Patient Characteristics	ESPB group	Control group	GA induction
Tulgar 2018 (1)	30 (15/15)	Laparoscopiccholecystectomy	I-II	18–65 years of age	Bilateral ultrasound-guided ESPB at the level of T9 transverse process using 10 mL of bupivacaine 0.375% on each side	Received no intervention	Propofol 2–3mgkg − 1,fentanyl100μg and rocuronium bromide 0.6 mg kg − 1
Gürkan 2018	50 (25/25)	Elective breast cancer surgery	I-II	Aged 20–65 years	Ultrasound (US)-guided ESPB with 20 ml 0.25% bupivacaine at the T4 vertebral level	Received no intervention	Propofol(2–3 mg kg − 1) and fentanyl(2 mg kg − 1) iv, rocuronium 0.6 mg kg − 1
Tulgar 2018 (2)	40 (20/20)	Hip and proximal femur surgery	I-III	Aged 18–65 years	Ultrasound-guided ESPB at T9 vertebrae level with 20 ml bupivacaine 0.5%, 10 ml lidocaine 2%,	Underwent thesame procedure but had no block	Propofol 2-3 mg/kg, fentanyl 100 μg and rocuronium bromide 0.6 mg/kg.
Singh 2019 (1)	40 (20/20)	Elective lumbarspine surgery	I-III	18–65 years of age	Ultrasound (US)-guided ESPB with total 20 ml 0.5% bupivacaine at the T10 vertebral level	Received no intervention	Propofol 2 to3 mg/kg, morphine 0.1 mg/kg and vecuronium 0.1 mg/kg
Gürkan 2019	50 (25/25)	Elective unilateral breast surgery	I-II	Aged 18–65 years	Ultrasound (US) guided ESP block with 20 ml 0.25% bupivacaine at the T4 vertebral level	Received no intervention	Propofol (2–3 mg kg − 1) and fentanyl (2 μg kg − 1) iv and rocuronium 0.6 mg kg − 1
Singh 2019 (2)	40 (20/20)	Modified radical mastectomy	I-II	Female patients between 20 and 55 years	Ultrasound (US)-guided ESP block with total 20 ml 0.5% bupivacaine at the T5 vertebral level	Received no intervention	Propofol 2–3 mg kg − 1 , morphine 0.1 mg kg – 1, and vecuronium 0.1 mg kg − 1
Aksu 2019 (1)	46 (23/23)	LaparoscopicCholecystectomy	I-II	20–75 years of age	Ultrasound (US) guided ESP block with 20 ml 0.25% bupivacaine at the T5–6 vertebral level	Received no intervention	Propofol (2–3 mg kg-1) and fentanyl (2 mg kg-1) iv and Rocuronium (0.6 mg kg-1)IV
Ciftci 2019	60 (30/30)	Video-Assisted Thoracic surgery	I-II	18–65 years of age	Ultrasound guided Bilateral ESP block with20ml of 0.375% bupivacaine at the T5 vertebral level	Received no intervention	Propofol (2–2.5 mg/kg) and fentanyl (1–1.5 mg/kg) and rocuronium bromide (0.6 mg/kg)
Ciftci 2019	60 (30/30)	Video-Assisted Thoracic surgery	I-II	18–65 years of age	Ultrasound guided Bilateral ESP block with20ml of 0.375% bupivacaine at the T5 vertebral level	Received no intervention	Propofol (2–2.5 mg/kg) and fentanyl (1–1.5 mg/kg) and rocuronium bromide (0.6 mg/kg)
Yayik 2019	60 (30/30)	Lumbar Spinal Decompression Surgery	I-III	18–65 years of age	Ultrasound guided Bilateral ESP block with 0.25% bupivacaine 20 mL at the L3 vertebral level	No intervention was performed	2 mg/kg IV propofo, 0.6 mg/kg IV rocuronium and 2 mcg/kg IV fentanyl
Hamed 2019	60 (30/30)	Abdominal hysterectomy	I-III	Women aged 40–70 years old and weighed 50–90 kg	Ultrasound-guided ESPB at T9 vertebrae level with 20 ml bupivacaine 0.5%.	Underwent the same procedure but had a sham injection(20 ml of saline)	Fentanyl 2 mcg.kg − 1 and propofol 2 mg.kg1, followed by atracurium 0.5 mg.kg − 1
AKSU 2019 (2)	50 (25/25)	elective breast surgery	I-II	Aged between 25 and 70 years	Ultrasound-guided ESPB betweenT2 and T4 with 10 ml of 0.25% bupivacaine	No intervention was performed	Propofol (2–3 mg/kg) and fentanyl (2 mg/kg) iv and Rocuronium 0.6 mg/kg was administered iv

### Primary outcomes

**Fig. 2:**
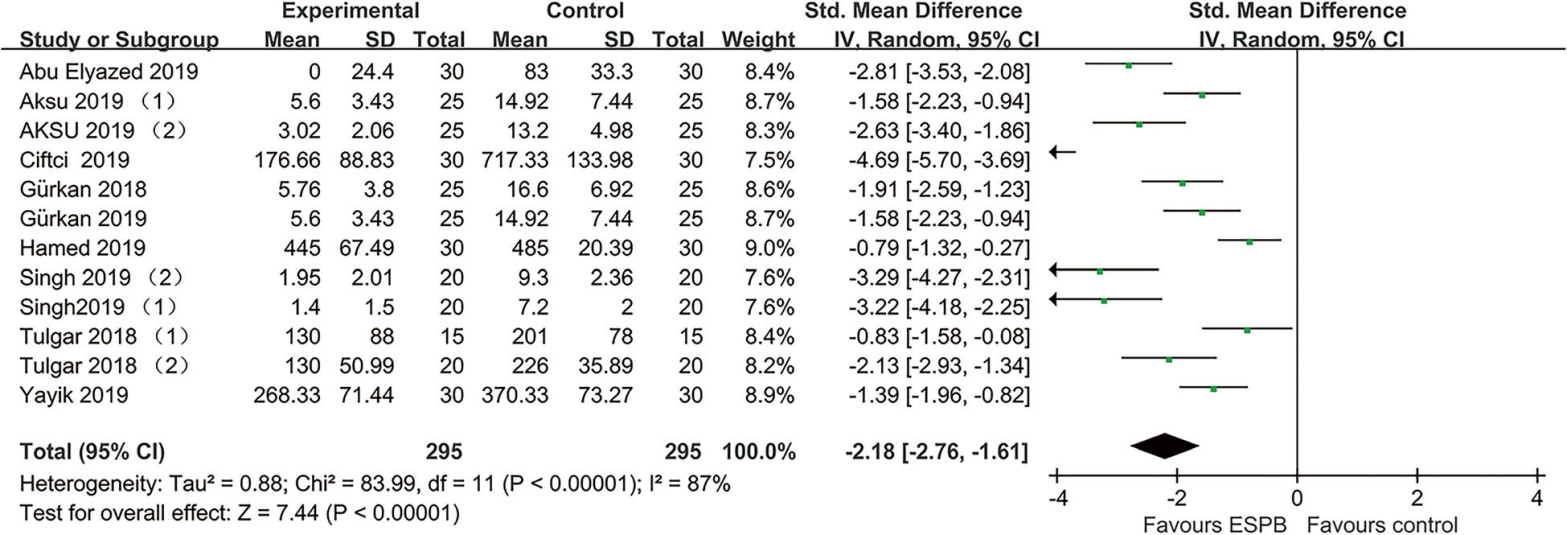
Forest plots of morphine consumption 24 h after surgery

**Fig. 3:**
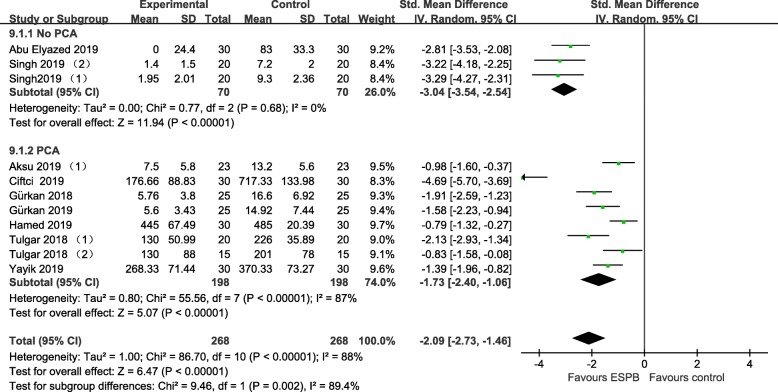
Forest plots of subgroup analysis

All RCTs [5–7, 17–25] reported data on intravenous opioid consumption 24 h after surgery. Pooled analysis showed that ultrasound-guided ESPB was associated with a reduction of opioid 24 h after surgery (− 2.18, 95% CI − 2.76 to − 1.61; *P* < .00001; Fig. 2). Substantial heterogeneity was observed among these studies (P for heterogeneity<.00001; I^2^ = 87%). The finding was consistent in subgroup analysis. (Fig. 3).

### Secondary outcomes

Ultrasound-guided ESPB significantly decrease pain scores at the 1 h(− 0.80, 95% CI − 1.54 to − 0.06;) and 6 h[− 0.64, 95% CI − 0.99 to − 0.30;).Furthermore, No. need rescue analgesia (0.41, 95% CI 0.25 to 0.66; *P* = .0002, I^2^ = 67%) was lower in the ESPB group and time to first rescue analgesic (4.56, 95% CI 1.89 to 7.22) was longer in the ESPB group. Pain scores at 12 h,24 h after surgery and PONV did not achieve statistical significant significance. All outcomes of the identified trials are reported in Table 2.

**Table 2:** Outcome data of RCTs included in the meta-analysis

Outcome	Studies include	RR or Std.mean differance [95%CI]	*P*-value for statistical significance	P-value for heterogeneity	I^2^ test for heterogeneity
Opiod consumption in the first 24 h (mg)	12	-2.18[−2.76,-1.61]	< 0.00001	< 0.00001	87%
VAS/NRS scores at the 1st hour	6	−0.80[−1.54,-0.06]	0.03	< 0.00001	88%
VAS/NRS scores at the 6th hour	8	−0.64[− 0.99,-0.30]	0.0003	0.03	58%
VAS/NRS scores at the 12th hour	6	−0.16[− 0.66,0.33]	0.51	0.0008	76%
VAS/NRS scores at the 24th hour	8	−0.83[−1.78,0.12]	0.09	0.00001	94%
Rescue analgesia requirement(n)	7	0.41 [0.25,0.66]	0.0002	0.006	67%
Time to first rescue analgesic (min)	3	4.56 [1.89,7.22]	0.0008	0.00001	95%
POVN(postoperative nausea and vomiting)	9	0.45 [0.20,1.00]	0.05	< 0.00001	84%

### Quality assessment and publication bias

**Fig. 4:**
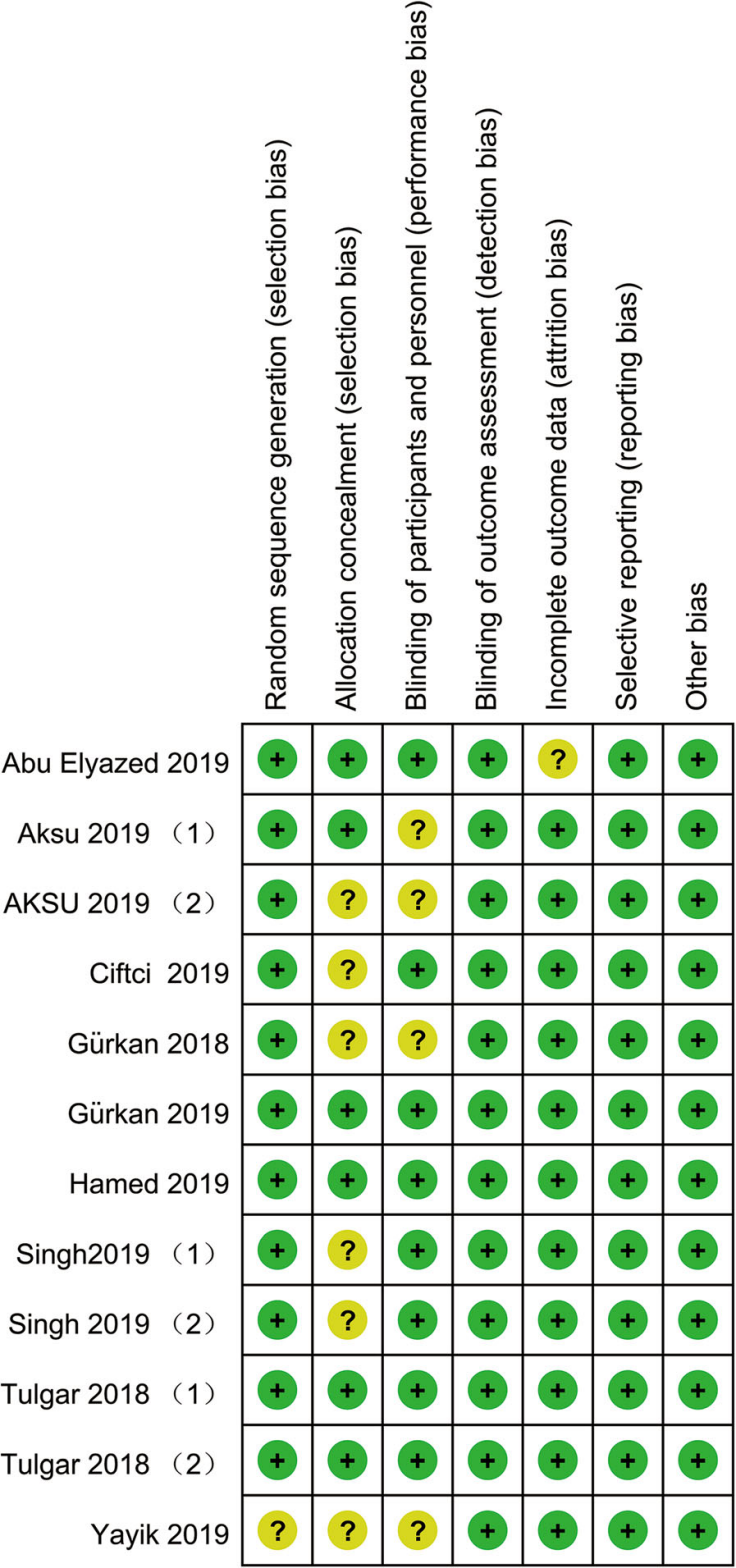
Risk of bias

**Fig. 5:**
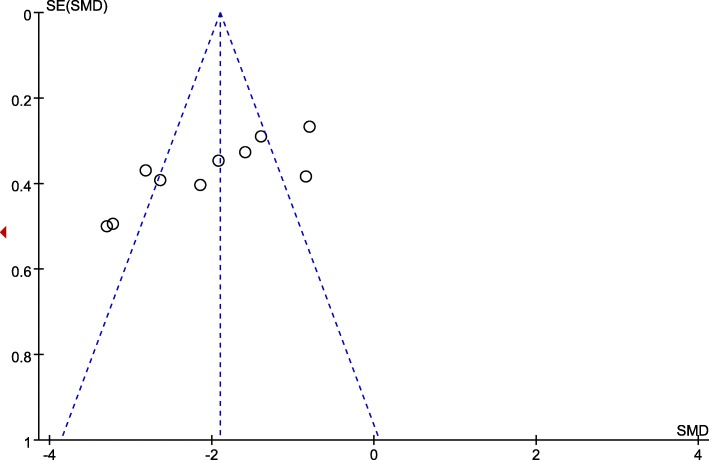
Funnel plot evaluating publication bias

Four trials at a low risk of bias, and 8 trials at an unclear risk of bias. The randomisation procedure was adequately generated in 11 trials [5–7, 17–20, 22–25]. Since we subjectively judge the outcome measurement was little prone to be changed by lacking of blinding, all RCTs included were classified as low risk of bias at blinding of outcome assessments. Assessment of risk-of-bias summary of all RCTs are presented in (Fig. 4). There was no evidence of publication bias by inspection of the funnel plot (Fig. 5).

## Discussion

### Main finding

The main finding of this meta-analysis is that ultrasound-guided ESPB significantly reduced opioid consumption 24 h after surgery. It further reduced pain scores and patients who need rescue analgesia, besides, it prolonged the time to first request of rescue analgesia. Despite of the high heterogeneity, the main finding was consistent in subgroup analyses.

### Possible mechanisms for findings

Ultrasound-guided ESPB is a peri-paravertebral regional anesthesia technique which is supposed to block the dorsal and ventral rami of the thoracic and abdominal spinal nerves [1], and thereby to block the anterior, posterior, and lateral thoracic and abdominal walls. However, the mechanisms of action and spread of LA are not fully elucidated. Several potential mechanisms have been posited. One of the suggested mechanisms of ultrasound-guided ESPB is paravertebral spread of LA, LA infiltration was observed from injection site to three vertebral levels cranially and four levels caudally [26]. Based on this mechanism, Coşarcan SK et al. [27] reported a modification ESPB and got good pain relief in various surgeries. However, the mechanism of paravertebral spread of LA remained debated in several cadaveric studies [28–30]. Another potential mechanism is epidural spread of LA. Schwartzmann A et al. [31], Tulgar S, et al. [32] and Altıparmak B, et al. [33] found unilateral erector spinae plane block result in bilateral sensory blockade in some patients, epidural spread of the LA during ESPB may explain this result. Moreover, some evidence indicated that penetration of LA acted on dorsal and ventral rami through the connective tissues and branch communication leaded to visceral analgesia [34, 35].

### Implications for clinical researches

Our findings demonstrated that ultrasound-guided ESPB was associated with a reduction of opioid consumption, which further proved the effectiveness of ESPB. However, ultrasound-guided ESPB has only been utilized in clinical setting for about 3 years, several important issues have not been resolved yet. First, the optimal concentration, volume and type of LA in ESPB is not well established. Although 20 and 30 ml of 0.25% bupivacaine or 0.5% ropivacaine were recommended [36], concentrations of 0.25–0.5% bupivacaine 10-20 ml were used in ultrasound-guided ESPB among all 12 RCTs included in this meta-analysis. Is bupivacaine more preferred than ropivacaine? why? We tried to make a judgment but stop by the insufficient evidence. More researches of ultrasound-guided ESPB on concentration, volume, type of LA are necessary. Next, although no complications of ultrasound-guided ESPB have been reported in all included RCTs, risks such as LA toxicity, vascular puncture and pneumothorax still need our attention. Two studies have reported pneumothorax associated with ESPB [37, 38], and Selvi O et al. [39] reported unintended motor block linked to ESPB. More complications may appear as the increased use of ultrasound-guided ESPB in population. Last, compared to other regional block techniques such as transversus abdominis plane block (TAPB), serratus plane block (SPB), and Quadratus Lumborum Block (QLB), is the erector spine block more effective in some operations where the block areas overlap? Several RCTs on these topics published recently but far from achieving convincing conclusions [40–42].

### Strengths and limitations

Our meta-analysis has several strengths. As far as we know, this is the first meta-analysis to evaluate the efficacy of ultrasound-guided ESPB in adults undergoing GA surgery. Besides, we performed this meta-analysis in compliance with the Cochrane Handbook and the PRISMA statement. Several notable limitations should be considered when interpreting the results. Firstly, the trials included have a modest sample size which could magnify the treatment effect. Secondly, the substantial heterogeneity was observed, one major factor result in heterogeneity is the diversity of surgery types (breast, lumbar spine, hip, abdominal etc). Parietal pain is more prominent in breast and lumbar spine, while visceral pain is the main component of postoperative pain following abdominal surgeries. The use of different types of opioid and supplementary analgesics such as paracetamol [23, 24] may also add an extra heterogeneity. Furthermore, owing to all patients were under GA surgeries, sensory blocking could not be evaluated adequately to exclude potential block failures of ESPB. Last, although we conducted a comprehensive literature search, it is hard to rule out the possibility of missing studies.

## Conclusion

In summary, ESPB block provides an effective analgesic in adults. However, the results should be interpreted cautiously since insufficient evidence, although accumulating. Further large-scale RCTs are required to support our results.

## Supplementary information

**Additional file 1.** PRISMA checklist

## Data Availability

Not applicable.

## Abbreviations

ESPB: Erector Spinae Plane Block
GA: General anesthesia
LA: Local anesthetic
RCTs: Randomized controlled trials
PRISMA: Preferred Reporting Items for Systematic Reviews and Meta Analyses
RCTs: American Society of Anesthesiologists
PONV: Postoperative nausea or vomiting
RRs: Relative risks
SD: Standard deviation
SMDs: Confidence intervals (CIs) standardized mean differences
PCA: Patient-controlled analgesia device

## References

[1] Forero M, Adhikary SD, Lopez H, Tsui C, Chin KJ (2016). The erector Spinae plane block: a novel analgesic technique in thoracic neuropathic pain. Reg Anesth Pain Med.

[2] El-Boghdadly K, Pawa A (2017). The erector spinae plane block: plane and simple. Anaesthesia.

[3] Chin KJ, Malhas L, Perlas A (2017). The erector Spinae plane block provides visceral abdominal analgesia in bariatric surgery: a report of 3 cases. Reg Anesth Pain Med.

[4] Bonvicini D, Tagliapietra L, Giacomazzi A, Pizzirani E (2018). Bilateral ultrasound-guided erector spinae plane blocks in breast cancer and reconstruction surgery. J Clin Anesth.

[5] Tulgar S, Kapakli MS, Senturk O, Selvi O, Serifsoy TE, Ozer Z (2018). Evaluation of ultrasound-guided erector spinae plane block for postoperative analgesia in laparoscopic cholecystectomy: a prospective, randomized, controlled clinical trial. J Clin Anesth.

[6] Tulgar S, Kose HC, Selvi O, Senturk O, Thomas DT, Ermis MN, Ozer Z (2018). Comparison of ultrasound-guided lumbar erector Spinae plane block and Transmuscular Quadratus Lumborum block for postoperative analgesia in hip and proximal femur surgery: a prospective randomized feasibility study. Anesth Essays Res.

[7] Singh S, Kumar G, Akhileshwar (2019). Ultrasound-guided erector spinae plane block for postoperative analgesia in modified radical mastectomy: a randomised control study. Ind J Anaesth.

[8] Moher D, Liberati A, Tetzlaff J, Altman DG (2009). Preferred reporting items for systematic reviews and meta-analyses: the PRISMA statement. BMJ.

[9] Higgins JPT, Green S (editors). Cochrane handbook for systematic reviews of interventions version 5.1.0 [updated March 2011]. The Cochrane Collaboration. 2011. Available from www.handbook.cochrane.org.

[10] Hozo SP, Djulbegovic B, Hozo I (2005). Estimating the mean and variance from the median, range, and the size of a sample. BMC Med Res Methodol.

[11] Higgins JP, Altman DG, Gotzsche PC, Juni P, Moher D, Oxman AD, Savovic J, Schulz KF, Weeks L, Sterne JA (2011). The Cochrane Collaboration's tool for assessing risk of bias in randomised trials. BMJ.

[12] Higgins JP, Thompson SG, Deeks JJ, Altman DG (2003). Measuring inconsistency in meta-analyses. BMJ.

[13] Krishna SN, Chauhan S, Bhoi D, Kaushal B, Hasija S, Sangdup T, Bisoi AK (2019). Bilateral erector Spinae plane block for acute post-surgical pain in adult cardiac surgical patients: a randomized controlled trial. J Cardiothorac Vasc Anesth.

[14] Macaire P, Ho N, Nguyen T, Nguyen B, Vu V, Quach C, Roques V, Capdevila X (2019). Ultrasound-guided continuous thoracic erector Spinae plane block within an enhanced recovery program is associated with decreased opioid consumption and improved patient postoperative rehabilitation after open cardiac surgery-a patient-matched, controlled before-and-after study. J Cardiothorac Vasc Anesth.

[15] Breebaart MB, Van Aken D, De Fre O, Sermeus L, Kamerling N, de Jong L, Michielsen J, Roelant E, Saldien V, Versyck B (2019). A prospective randomized double-blind trial of the efficacy of a bilateral lumbar erector spinae block on the 24h morphine consumption after posterior lumbar inter-body fusion surgery. Trials.

[16] Urits I, Charipova K, Gress K, Laughlin P, Orhurhu V, Kaye AD, Viswanath O (2019). Expanding role of the erector Spinae plane block for postoperative and chronic pain management. Curr Pain Headache Rep.

[17] Singh S, Choudhary NK, Lalin D, Verma VK. Bilateral ultrasound-guided erector Spinae plane block for postoperative analgesia in lumbar spine surgery: a randomized control trial. J Neurosurg Anesthesiol 2019. 10.1097/ANA.0000000000000603 31033625

[18] Ciftci B, Ekinci M, Celik EC, Tukac IC, Bayrak Y, Atalay YO. Efficacy of an ultrasound-guided erector Spinae plane block for postoperative analgesia management after video-assisted thoracic surgery: a prospective randomized study. J Cardiothorac Vasc Anesth. 2019. 10.1053/j.jvca.2019.04.026 31122843

[19] Gurkan Y, Aksu C, Kus A, Yorukoglu UH (2019). Erector spinae plane block and thoracic paravertebral block for breast surgery compared to IV-morphine: a randomized controlled trial. J Clin Anesth.

[20] Hamed MA, Goda AS, Basiony MM, Fargaly OS, Abdelhady MA (2019). Erector spinae plane block for postoperative analgesia in patients undergoing total abdominal hysterectomy: a randomized controlled study original study. J Pain Res.

[21] Yayik AM, Cesur S, Ozturk F, Ahiskalioglu A, Ay AN, Celik EC, Karaavci NC (2019). Postoperative analgesic efficacy of the ultrasound-guided erector Spinae plane block in patients undergoing lumbar spinal decompression surgery: a randomized controlled study. World Neurosurg.

[22] Aksu C, Kuş A, Yörükoğlu HU, Kılıç CT, Gürkan Y (2019). The effect of erector Spinae plane block on postoperative pain following laparoscopic cholecystectomy: a randomized controlled study. JARSS.

[23] Gurkan Y, Aksu C, Kus A, Yorukoglu UH, Kilic CT (2018). Ultrasound guided erector spinae plane block reduces postoperative opioid consumption following breast surgery: a randomized controlled study. J Clin Anesth.

[24] Abu Elyazed MM, Mostafa SF, Abdelghany MS, Eid GM (2019). Ultrasound-guided erector Spinae plane block in patients undergoing open Epigastric hernia repair: a prospective randomized controlled study. Anesth Analg.

[25] Aksu C, Kus A, Yorukoglu HU, Tor Kilic C, Gurkan Y (2019). Analgesic effect of the bi-level injection erector spinae plane block after breast surgery: A randomized controlled trial. Agri.

[26] Chin KJ, Adhikary S, Sarwani N, Forero M (2017). The analgesic efficacy of pre-operative bilateral erector spinae plane (ESP) blocks in patients having ventral hernia repair. Anaesthesia.

[27] Cosarcan SK, Gurkan Y, Dogan AT, Ercelen O (2020). Targeted modification of erector spinae plane block. Acta Anaesthesiol Scand.

[28] Ivanusic J, Konishi Y, Barrington MJ (2018). A cadaveric study investigating the mechanism of action of erector Spinae blockade. Reg Anesth Pain Med.

[29] Aponte A, Sala-Blanch X, Prats-Galino A, Masdeu J, Moreno LA, Sermeus LA (2019). Anatomical evaluation of the extent of spread in the erector spinae plane block: a cadaveric study. Can J Anaesth.

[30] Yang HM, Choi YJ, Kwon HJ, Cho TH, Kim SH, O J (2018). Comparison of injectate spread and nerve involvement between retrolaminar and erector spinae plane blocks in the thoracic region: a cadaveric study. Anaesthesia.

[31] Schwartzmann A, Peng P, Maciel MA, Forero M (2018). Mechanism of the erector spinae plane block: insights from a magnetic resonance imaging study. Can J Anaesth.

[32] Tulgar S, Selvi O, Ahiskalioglu A, Ozer Z (2019). Can unilateral erector spinae plane block result in bilateral sensory blockade?. Can J Anaesth.

[33] Altiparmak B, Korkmaz Toker M, Uysal AI (2020). Potential mechanism for bilateral sensory effects after unilateral erector spinae plane blockade in patients undergoing laparoscopic cholecystectomy. Can J Anaesth.

[34] Hamilton DL, Manickam B (2017). Erector spinae plane block for pain relief in rib fractures. Br J Anaesth.

[35] Adhikary SD, Bernard S, Lopez H, Chin KJ (2018). Erector Spinae plane block versus Retrolaminar block: a magnetic resonance imaging and anatomical study. Reg Anesth Pain Med.

[36] Krishnan S, Cascella M: Erector Spinae plane block. In: StatPearls. Edn. Treasure Island (FL): StatPearls Publishing StatPearls Publishing LLC.; 2020. 31424889

[37] Ueshima H (2018). Pneumothorax after the erector spinae plane block. J Clin Anesth.

[38] Hamilton DL (2019). Pneumothorax following erector spinae plane block. J Clin Anesth.

[39] Selvi O, Tulgar S (2018). Ultrasound guided erector spinae plane block as a cause of unintended motor block. Rev Esp Anestesiol Reanim.

[40] Altiparmak B, Korkmaz Toker M, Uysal AI, Kuscu Y, Gumus Demirbilek S (2019). Ultrasound-guided erector spinae plane block versus oblique subcostal transversus abdominis plane block for postoperative analgesia of adult patients undergoing laparoscopic cholecystectomy: randomized, controlled trial. J Clin Anesth.

[41] Aksu C, Sen MC, Akay MA, Baydemir C, Gurkan Y (2019). Erector Spinae plane block vs Quadratus Lumborum block for pediatric lower abdominal surgery: a double blinded, prospective, and randomized trial. J Clin Anesth.

[42] Gaballah KM, Soltan WA, Bahgat NM (2019). Ultrasound-guided Serratus plane block versus erector Spinae block for postoperative analgesia after video-assisted Thoracoscopy: a pilot randomized controlled trial. J Cardiothorac Vasc Anesth.

