# Supplementary figures and images for: Ultrasound-guided erector spinae plane block for postoperative analgesia: a meta-analysis of randomized controlled trials
Source: BMC Anesthesiol. 2020 Apr 14;20:83. doi: 10.1186/s12871-020-00999-8 (PMC7155251; doi:10.1186/s12871-020-00999-8)

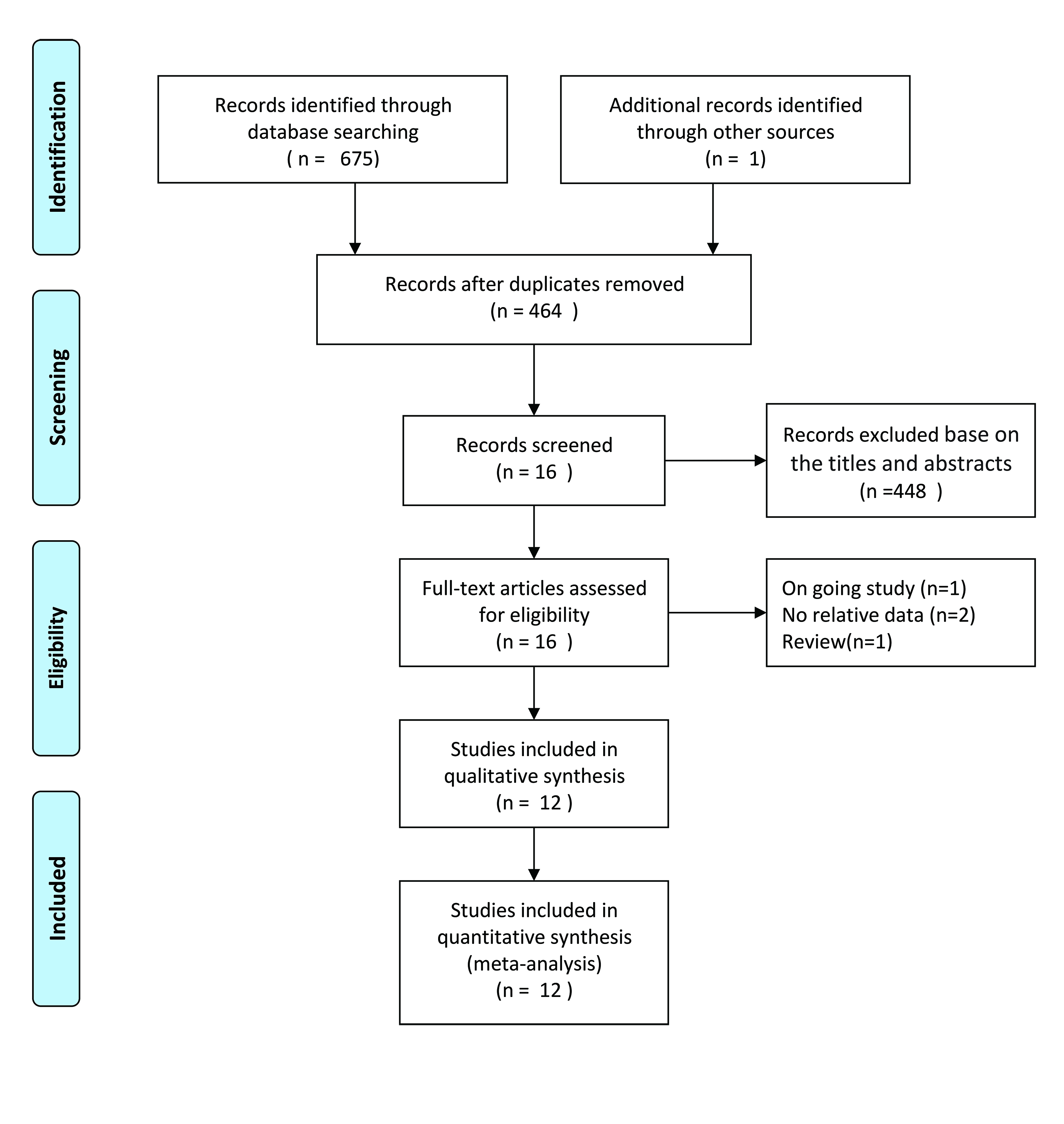

Supplement: Supplementary file 1 — Additional file 1. PRISMA checklist [file 12871_2020_999_MOESM1_ESM.tif]
